# Prolonged Corticosteroid Use Leading to Cerebral Venous Sinus Thrombosis: A Rare Association

**DOI:** 10.7759/cureus.41104

**Published:** 2023-06-28

**Authors:** Vikram Jain, Jaideep Pilania, Mayank Agarwal, Nonita Thokchom, Monika Pathania

**Affiliations:** 1 Internal Medicine, All India Institute of Medical Sciences, Rishikesh, IND

**Keywords:** cerebral venous sinus thrombosis, corticosteroids, young male, venous, cerebral sinus, thrombosis

## Abstract

Partial or complete obstruction of blood flow in venous sinuses of the brain leads to a clinical condition termed cerebral venous sinus thrombosis (CVST). In most diagnosed cases, CVST has at least one risk factor identified among many postulated, and it most commonly includes acquired or inherited prothrombotic conditions. Steroid intake for intentional weight gain is prevalent in the general population, especially among new-generation bodybuilders and athletes. Excess exogenous steroids have many adverse effects, and increasing the risk of venous thromboembolism is one of them. The role of these steroids in developing CVST among such individuals has not been reported frequently in the literature. We report a case of a young male with a history of chronic exogenous steroid intake to increase his body weight, who presented with clinical features of CVST.

## Introduction

Partial or complete obstruction of blood flow in venous sinuses of the brain leads to cerebral venous sinus thrombosis (CVST) [1]. Though it is an uncommon entity, more than 85% of diagnosed cases of CVST have at least one risk factor identified among them, most commonly acquired or inherited prothrombotic condition [2]. It is known for a very long time that steroids are misused to gain body weight in the general population, especially among bodybuilders and athletes [3]. Excess exogenous steroids increase the risk of venous thromboembolism [4]. The role of these steroids in developing CVST among such individuals has not been reported frequently in the literature.

## Case presentation

Case history

A 22-year-old young male studying in college, with a history of consumption of 8 mg per day dexamethasone from a local chemist without any prescription for the past three years to gain weight presented with insidious onset, progressive, moderate intensity, continuous, non-radiating, holocranial, throbbing headache for the past one-month duration. He also complained of insidious onset, progressive, painless blurring of vision in both eyes for the past seven days. There was no history of alcohol or substance abuse, smoking, diabetes, or other systemic diseases.

**Figure 1:**
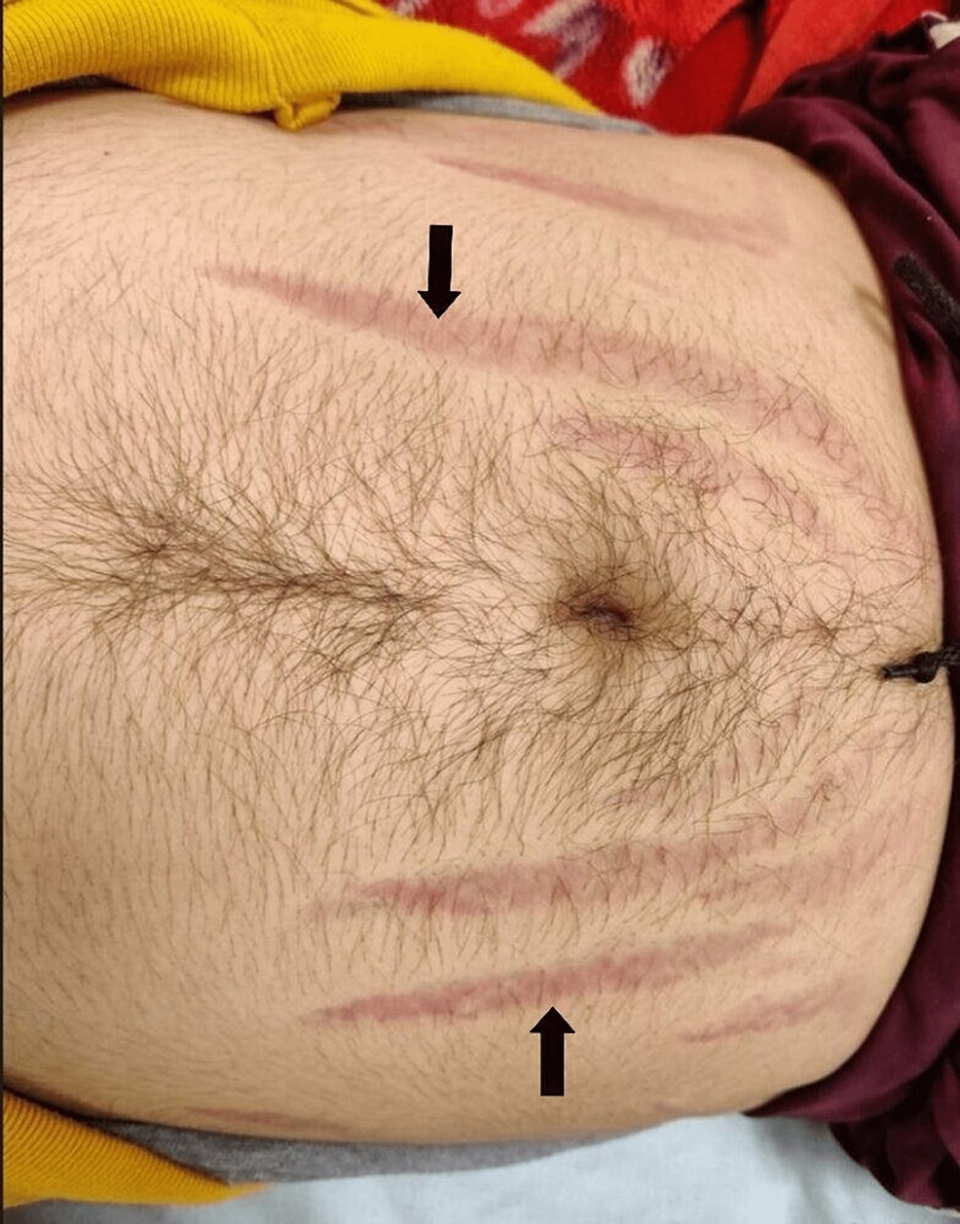
Longitudinal, violaceous stretch marks over the abdomen (black arrows).

On physical examination, longitudinal violaceous stretch marks over his abdomen were noted (Figure 1).

**Figure 2:**
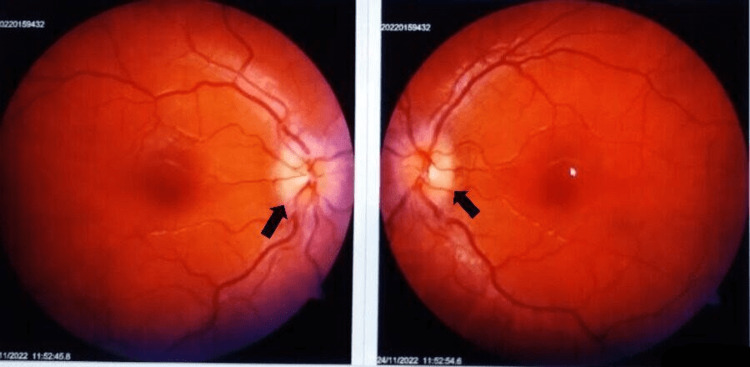
Fundus photograph showing papilledema in both eyes (black arrows).

On admission, he had a pulse rate of 92 bpm, blood pressure of 108/74 mm Hg, respiratory rate of 14 breaths per minute, and SpO2 of 99% on room air. Body mass index was 25.7 kg/m2. The patient was conscious and oriented. The remainder of the general physical examination was unremarkable, and no other signs and symptoms related to Cushing's syndrome were noted. On a detailed neurological examination, he had visual acuity of 6/30 in both eyes. All cranial nerves were normal on examination, and all brain stem functions were preserved. There were no bladder or bowel disturbances. Muscle strength was normal in all four limbs, and deep tendon reflexes of +2 were noted in all four limbs. The sensory examination was normal. Complete blood counts, erythrocyte sedimentation rate, C-reactive protein, serum electrolytes, and liver and kidney functions were within normal limits (Table 1). Fundoscopy showed grade 2 papilledema in both eyes (Figure 2).

**Table 1:** Summary of blood tests on admission.

Blood tests	Value	Reference range
Hemoglobin (mg/dl)	12.7	13-17 g/dL
White cell count (10^3/uL)	6.68	4-11 10^3/uL
Platelets (10^3/uL)	342,000	150-400 10^3/uL
Alanine aminotransferase (IU/L)	61	0 - 50 U/L
International normalized ratio	1.10	
Fibrinogen (g/L)	3.89	2.00-4.00 (g/l)
D-dimer (mg/L)	0.2	<0.5 (mg/L FEU)
Protein S	82%	(60-140%)
Protein C	100%	(70-140%)
Antithrombin III	88%	(80-120%)
Factor V Leiden mutation	Negative	
Prothrombin gene mutation	Negative	
Antinuclear antibody	Negative	
Lupus anticoagulant	Negative	
Anticardiolipin IgG (GPL)	7.6	(<15)
Anti-beta-2-glycoprotein-1 antibody	1.04	(<20)

**Figure 3:**
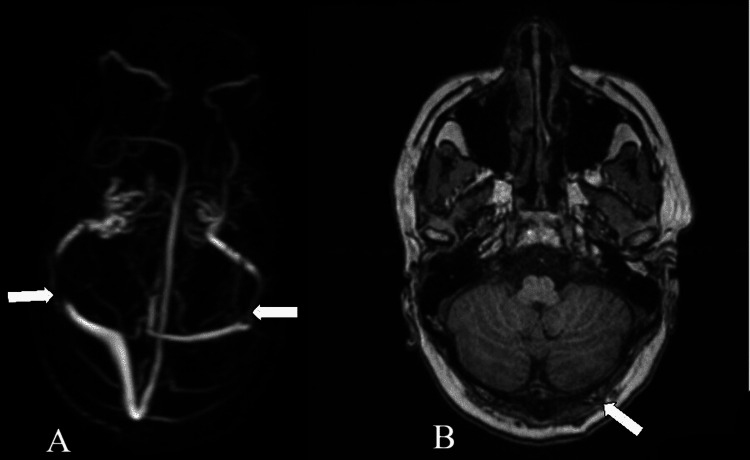
(A) Magnetic resonance venography showing a filling defect in the right transverse-sigmoid sinus junction and left transverse-sigmoid sinus junction showing luminal stenosis shown by white arrows. (B) Axial T1-weighted image showing increased signal demonstrating thrombosis in the left and right transverse-sigmoid sinus junction shown by the white arrow.

**Figure 4:**
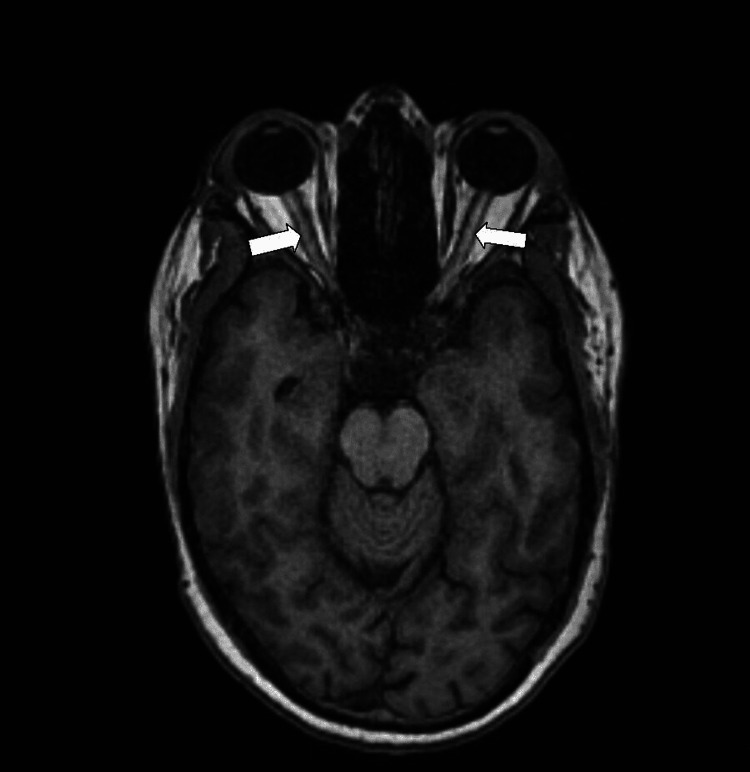
Axial T1-weighted image showing bilateral optic nerve heads flattened and distended bilateral optic nerve sheaths shown by white arrows.

Contrast-enhanced MRI of the brain with magnetic resonance (MR) venography showed a linear filling defect in the right transverse sigmoid junction (for a length of 20 mm on the right) and left transverse sigmoid junction showing luminal stenosis (Figure 3); bilateral optic nerve heads were also flattened. The bilateral optic nerve sheath was distended with CSF, suggestive of benign intracranial hypertension (Figure 4).

The patient was started on therapeutic anticoagulation with low molecular weight heparin 0.6 mg subcutaneous twice daily dosing. For further evaluation of venous thrombosis, a thrombophilia profile was sent, the results of which were normal (Table 1).

After five days of low molecular weight heparin, the patient continued oral anticoagulant therapy with rivaroxaban 20 mg tablet once daily. His headache declined significantly after three days of low molecular weight heparin injections, and visual acuity improved to 6/18 in both eyes after seven days. The patient was discharged after seven days on rivaroxaban tablet initially for at least three months course. On follow-up, repeated investigations, including thrombophilia workup, showed no significant abnormality suggesting the role of long-term steroid therapy in the development of CVST in our patient.

## Discussion

CVST is a rare disease that has various etiological factors, including prothrombotic conditions, which are either genetic, like antithrombin deficiency, protein C deficiency or protein S deficiency, factor V Leiden deficiency, and hyperhomocysteinemia, or acquired, including obesity, oral contraceptives, testosterone replacement therapy, pregnancy, and the puerperium, malignancies, celiac disease, and infections like COVID-19 in recent times. The underlying cause is still not identified in approximately 15% of patients [2]. The risk of venous thromboembolism (VTE) with the use of corticosteroids is not well documented. Sigrun A. Johannesdottir et al., in a nationwide study, showed an increased risk of VTE in patients taking corticosteroids regularly for more than 90 days of duration with an incidence rate ratio (IRR) of 1.98 [4]. Cushing's syndrome, which is a state of high endogenous cortisol, is reported to be a hypercoagulable state. Although the pathogenesis is not well documented, increased Von Willebrand factor, impaired fibrinolytic activity, and shortened activated partial thromboplastin time (aPTT) are usually found among patients taking long-term corticosteroids [5].

The annual incidence of CVST in various studies ranges from 1.32 to 1.57 per 100,000 population [6], more common in the younger population and women than in men [7]. The clinical presentation of CVST is broadly divided into three distinct clinical syndromes: isolated intracranial hypertension (headache, papilledema, and visual disturbances), focal syndrome like seizures (39.3% cases), and paresis (37.2% cases) and encephalopathy in the form of altered mental status and coma [8]. MR venography and CT venography have very high sensitivity and specificity to diagnose CVST. Cerebral digital subtraction angiography is the gold standard to diagnose this condition, although routinely not used and reserved where despite high clinical suspicion, CT or MR venography is inconclusive. Long-term anticoagulation is advised with the aim to prevent cerebral vein and sinus thrombosis (CVT) recurrence, which affects 2-7% of patients, and to prevent extracerebral venous thrombosis, which occurs in up to 5% of patients with CVT [2]. Warfarin or newer oral anticoagulants are preferred after the acute phase in the treatment of CVST with the aim of maintaining an international normalized ratio between 2 and 3 if warfarin is used.

The lifetime use of steroids in the general population is estimated to be around 1-5% [9]. Steroids are also used in therapeutic doses for long duration in various autoimmune diseases [10], but the occurrence of steroid-induced CVST is not widely reported. In our case, the absence of known inflammatory disease, history of smoking, alcohol use, and diabetes, absence of any finding suggesting dehydration, absence of any known infective etiology, and normal tests for thrombophilia lead to the exclusion of all other etiologies of CVST. After giving three months of oral anticoagulant, based on repeat MRI and symptom analysis, long-term oral anticoagulant therapy is planned.

## Conclusions

In conclusion, after ruling out all the known etiologies of CVST by history, clinical examination, and laboratory investigations, and with no history of lumbar puncture, we can suggest that chronic steroid use may be a risk factor for CVST. However, further research is warranted to look for possible pathophysiology.
